# Enhancing predictions of antimicrobial resistance of pathogens by expanding the potential resistance gene repertoire using a pan-genome-based feature selection approach

**DOI:** 10.1186/s12859-022-04666-2

**Published:** 2022-04-15

**Authors:** Ming-Ren Yang, Yu-Wei Wu

**Affiliations:** 1 Graduate Institute of Biomedical Informatics, College of Medical Science and Technology, Taipei Medical University, 250 Wuxing St., Sinyi District, Taipei, 11031 Taiwan; 2 Department of Electrical Engineering, National Taiwan University of Science and Technology, Taipei, 106 Taiwan; 3 Clinical Big Data Research Center, Taipei Medical University Hospital, Taipei, 11031 Taiwan

**Keywords:** Antimicrobial resistance, Pan-genome, Feature selection, eXtreme gradient boosting, XGBoost, Hypothetical proteins

## Abstract

**Background:**

Predicting which pathogens might exhibit antimicrobial resistance (AMR) based on genomics data is one of the promising ways to swiftly and precisely identify AMR pathogens. Currently, the most widely used genomics approach is through identifying known AMR genes from genomic information in order to predict whether a pathogen might be resistant to certain antibiotic drugs. The list of known AMR genes, however, is still far from comprehensive and may result in inaccurate AMR pathogen predictions. We thus felt the need to expand the AMR gene set and proposed a pan-genome-based feature selection method to identify potential gene sets for AMR prediction purposes.

**Results:**

By building pan-genome datasets and extracting gene presence/absence patterns from four bacterial species, each with more than 2000 strains, we showed that machine learning models built from pan-genome data can be very promising for predicting AMR pathogens. The gene set selected by the eXtreme Gradient Boosting (XGBoost) feature selection approach further improved prediction outcomes, and an incremental approach selecting subsets of XGBoost-selected features brought the machine learning model performance to the next level. Investigating selected gene sets revealed that on average about 50% of genes had no known function and very few of them were known AMR genes, indicating the potential of the selected gene sets to expand resistance gene repertoires.

**Conclusions:**

We demonstrated that a pan-genome-based feature selection approach is suitable for building machine learning models for predicting AMR pathogens. The extracted gene sets may provide future clues to expand our knowledge of known AMR genes and provide novel hypotheses for inferring bacterial AMR mechanisms.

**Supplementary Information:**

The online version contains supplementary material available at 10.1186/s12859-022-04666-2.

## Background

The discovery and development of antibiotic drugs allowed people to explore the world more safely. Wound healing, joint replacement, and any type of open wounds/bacterial infections can be controlled very effectively with antibiotic drugs. However, the prevalence and misuse/abuse of antibiotics have also resulted in the emergence of drug resistance [termed antimicrobial resistance (AMR)] possessed by certain bacterial strains. As of today, resistance has been observed with virtually every antibiotic drug that has ever been developed [1]. It was also estimated that the death rate from hip replacements could increase from 0% to up to 30% if this condition continues to worsen [2], and the World Bank has warned that the annual financial costs of uncontrolled AMR may run to US$3.4 trillion by 2030 [3]. Therefore, controlling the use of antibiotic drugs is essential for preventing the worst case from occurring, and it is thus necessary to know or predict which antibiotic drugs are most effective for patients in order to prevent drug misuse.

Thanks to the development of mature next-generation sequencing (NGS) technology, sequencing and determining bacterial genomes are much easier than ever before. Several attempts have been made to predict AMR pathogens using genomic information. For example, Clausen et al. identified known genes related to AMR activities and used that genetic information to find AMR strains among 74 *Escherichia coli* and 69 *Klebsiella pneumoniae* isolates [4]. Similar approaches were also adopted to identify AMR strains from *Staphylococcus aureus* [5], *Pseudomonas aeruginosa* [6], and *Salmonella enterica* [7]. Other approaches, including nucleotide k-mer-based prototyping [8–10], amino acid composition [11], a population graph-based approach [12], single-nucleotide polymorphisms (SNPs) [13, 14], and Hidden Markov model (HMM)-based methods [15] were also developed and implemented for better prediction and identification of AMR strains based on their genomic sequences. Several software tools were also developed for predicting both AMR genes and strains, including CARD/RGI [16], ResFinder [17], ARIBA [18], KmerResistance [17], SRST2 [13], PointFinder [14], etc. The availability of such methodologies or tools may facilitate our understanding of AMR activities and provide more-accurate predictions of AMR pathogens.

Pan-genome, a concept that comprises different strains of the same microbial species, is a very powerful and convenient tool for describing similarities and differences among genetic contents of strains. In a nutshell, pan-genome is “made up of the sum of core and dispensable genomes,” as previously described [19]. In the beginning, pan-genomes were mainly used for describing prokaryotic species [19, 20]; most recently, however, the idea of a pan-genome has been extended to eukaryotes, including humans [21, 22], other animals [23, 24], and plants [25]. The pan-genome idea was also applied to antimicrobial analyses and predictions. For example, Scoary, a tool for the rapid scoring of genes in microbial pan-genomes, identified genes responsible for linezolid resistance in *S. epidermidis* [26]. Benchmarking on simulated *Streptococcus pneumoniae* genome datasets, as reported by Scoary, indicated that the performance of Scoary is dependent on the sample size, in which it is capable of reaching an 80% recall rate with a sample size of > 100*.* Another study conducted on *E. coli* found that the pan-genome gene content was more useful for predicting AMR strains than were SNPs [27]. We also applied a machine learning approach to the *E. coli* pan-genome and found that a subset of AMR genes was able to achieve a much-higher prediction accuracy [28], in which the genetic algorithm-based machine learning approach reached 95% accuracy for the selected AMR gene subset. Those studies clearly demonstrated the wide and plausible adoption of pan-genome ideas in AMR classification tasks and the importance in selecting crucial genes for better prediction of AMR mechanisms.

In this study, we attempted to uncover genes (including novel ones or even those without functional annotations) that were significantly related to AMR activities. By constructing pan-genomes from thousands of genomic sequences, we not only showed that a couple of genes selected by machine learning feature selection algorithms achieved much better prediction accuracies than known AMR genes, but also demonstrated that the majority of identified genes have unknown functions, and only a few of those selected genes are known AMR genes. Through this work, we showed the importance of continual mining of the functionalities of hypothetical genes and their potential relationships with AMR pathogens.

## Results

### Bacterial pan-genomes

**Fig. 1:**
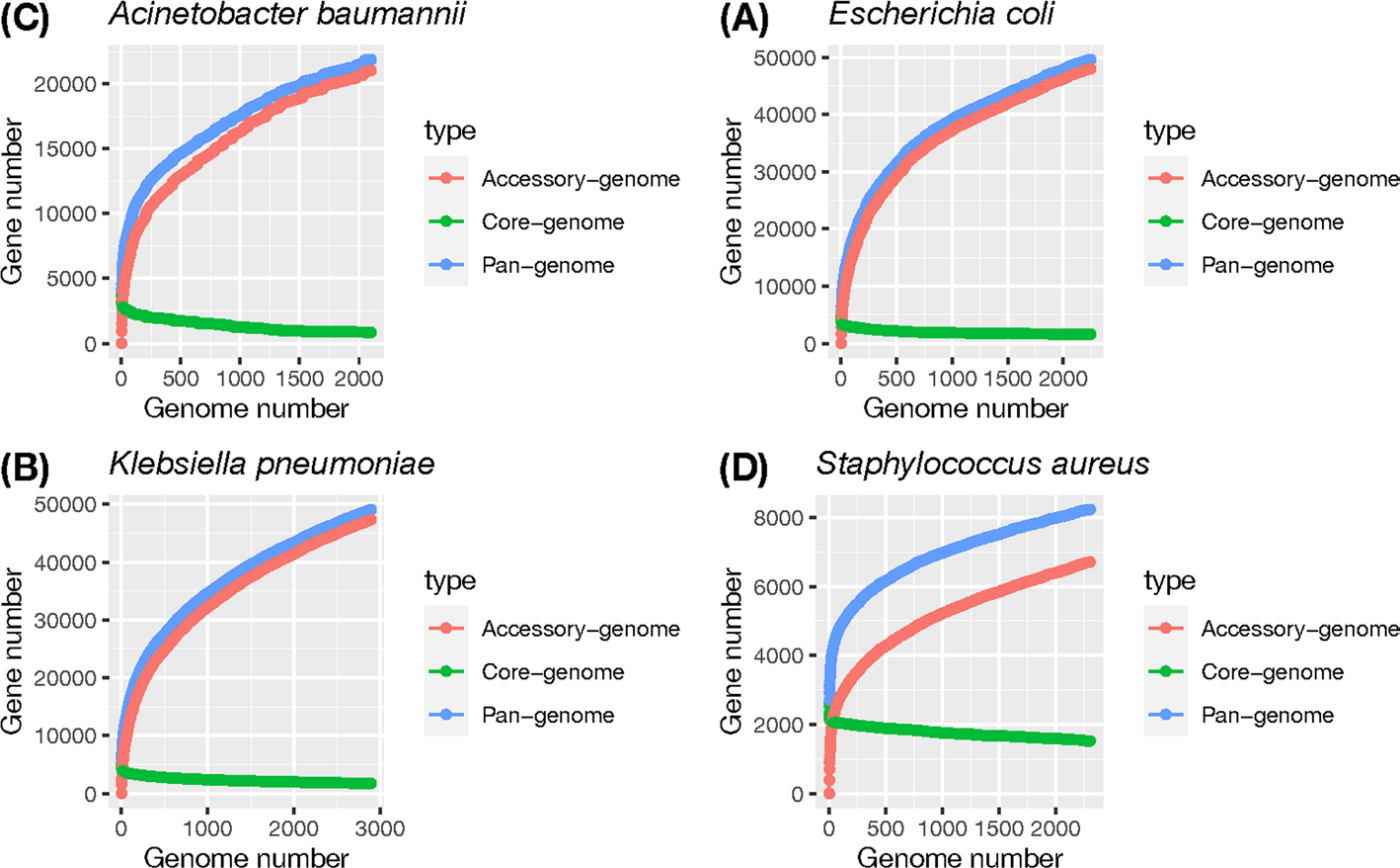
Pan-, core-, and accessory-genome growth curves of the four bacterial species, including **A** *Acinetobacter baumannii*, **B** *Escherichia coli*, **C** *Klebsiella pneumoniae*, and **D** *Staphylococcus aureus*. The x-axis indicates the number of genomes (strains), while the y-axis represents the number of gene clusters

Pan-genomes were built to analyze four bacterial species (viz., *Acinetobacter baumannii*, *E. coli*, *K. pneumoniae*, and *S. aureus*) with similar numbers of strains (all between 2000 and 3000). The numbers of strains, identified gene clusters, and numbers of core genes and accessory genes are listed in Table 1. As shown in Fig. 1, one can easily observe that the numbers of gene clusters for *A. baumannii* and *S. aureus* were significantly lower than those for the other two species despite similar strain numbers, indicating that the genetic diversities of *E. coli* and *K. pneumoniae* may be higher than those of the other two species.

**Table 1:** Pan-genome statistics of the four bacterial species

Species	No. of strains	No. of gene clusters	No. of core genes	No. of acc. genes
*Acinetobacter baumannii*	2,101	21,876	851	21,876
*Escherichia coli*	2,247	49,634	1,593	48,041
*Klebsiella pneumoniae*	2,895	49,104	1,730	47,374
*Staphylococcus aureus*	2,305	8,228	1,522	6,706

*acc.* accessory

Despite differences in gene cluster numbers, the analysis of the pan-genome growth curves suggested that all four species belong to open pan-genomes, indicating unlimited gene pools of all four species. By investigating the pan-genome distribution of the four species and fitting them to Heaps’ law distribution, we identified that the fitted $\gamma$ values were all > 0 (respective $\gamma$ values of *A. baumannii*, *E. coli*, *K. pneumoniae*, and *S. aureus* were 0.262, 0.311, 0.325, and 0.172; see Methods for details), suggesting that the four pan-genomes are all open pan-genomes. One can also observe that the pan-genome curves of the four bacteria were not flattened at all, consistent with the fitting results of Heaps’ law. The somewhat lower $\gamma$ values of *A. baumannii* and *S. aureus* also reflected the aforementioned observation that the gene diversities of these two species were significantly lower than those of *E. coli* and *K. pneumoniae*.

### Predicting AMR phenotypes using gene sets

**Fig. 2:**
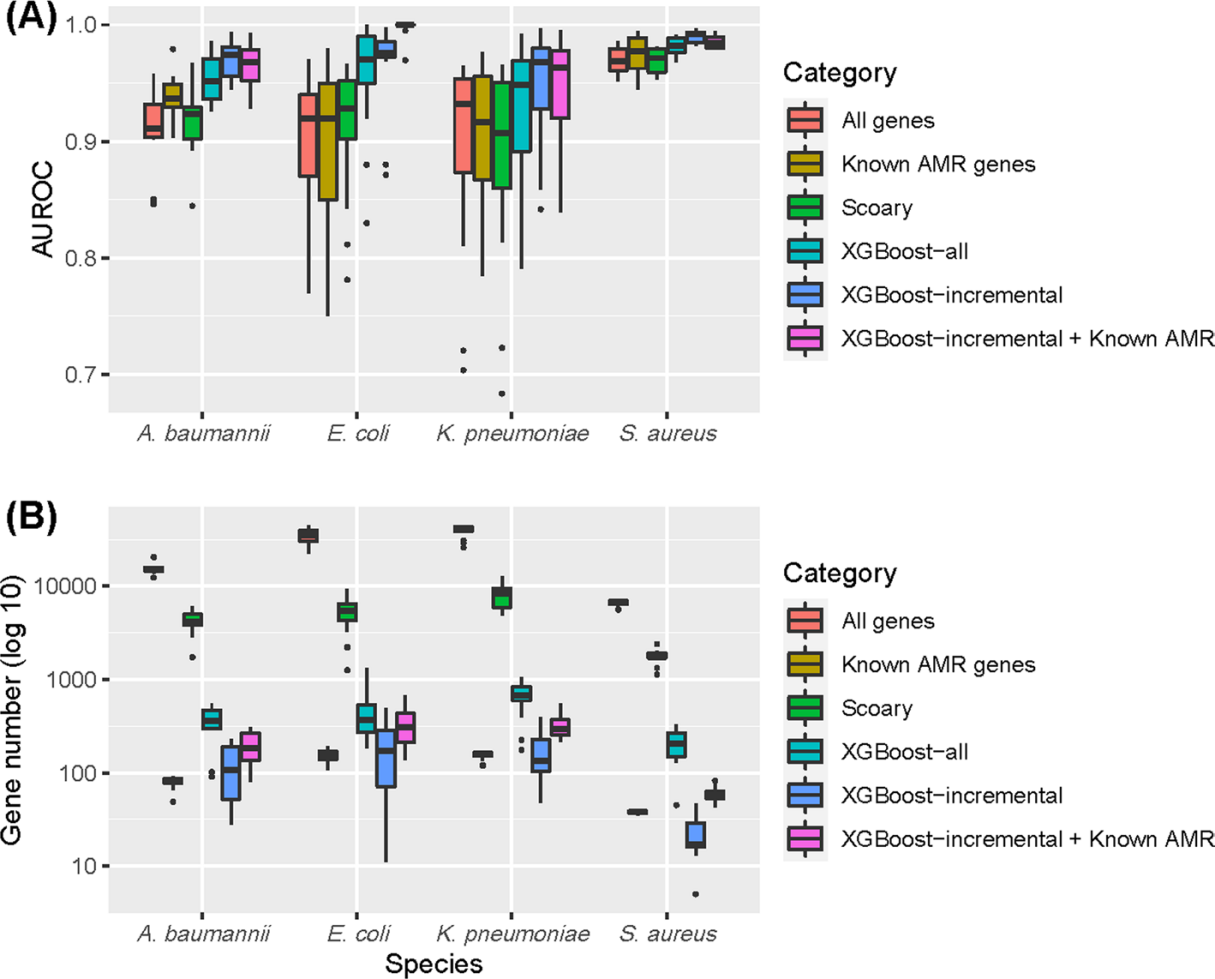
Boxplots indicating prediction accuracies and numbers of genes of different gene sets for antimicrobial resistance (AMR) prediction problems. **A** The AMR prediction accuracies of different gene sets were evaluated in terms of the area under the receiver operating characteristic (AUROC) curve. **B** The number of genes used in predicting AMR activities for different gene sets. The gene sets included: (1) all genes; (2) known AMR genes selected using CARD/RGI; (3) genes predicted by Scoary; (4) all genes selected using the XGBoost feature selection; (5) genes selected by the incremental approach on top of the XGBoost-selected genes; and (6) the combined set of genes selected by the incremental method and known AMR genes. The y-axis is the prediction accuracies in terms of the AUROC curve

Gene presence/absence tables for different antibiotic drug resistances were built for each bacterial species (see Methods for details). The numbers of drugs enrolled in our analysis were 10, 17, 13, and 8 for *A. baumannii*, *E. coli*, *K. pneumoniae*, and *S. aureus*, respectively (complete lists of enrolled drugs of the four species are given in Additional file 1: Tables S1–S4). After building different support vector machine (SVM) models for each drug resistance table and evaluating their prediction performances, we found that selecting relevant genes (features) using eXtreme Gradient Boosting (XGBoost) yielded better prediction performances. As shown in Fig. 2, XGBoost-selected genes (termed “XGBoost-all” in Fig. 2) clearly outperformed “all genes,” “known AMR genes,” and “Scoary-selected gene sets” in terms of prediction accuracy, indicating the ability of the XGBoost algorithm to select appropriate features for enhancing prediction performances. We noted that the prediction performances shown in Fig. 2 were estimated from distinct SVM classifiers, in which one classifier corresponded to one drug-resistance profile of one of the four species. Different gene sets also corresponded to different classifiers. The performances of the classifiers were then estimated using tenfold stratified cross validation (See Methods for details).

To further identify genes more relevant to AMR phenotypes and enhance prediction performances, an incremental approach was designed to select the best feature set among XGBoost-selected features. The core idea of the incremental approach is to pick genes sorted by feature importance values, one-by-one cumulatively, and then calculate the model prediction performances of the selected genes in order to find the subset with the best outcome. As shown in Fig. 2, genes picked by the incremental approach (termed “XGBoost-incremental” in Fig. 2) achieved the overall best performance (overall > 95% area under the receiver operating characteristic (AUROC) curve) with the most succinct gene sets (mostly < 100). The results indicated that choosing the most plausible gene set is indeed capable of significantly enhancing the prediction performances of AMR prediction problems. Evaluating gene sets using other prediction performance metrics, including precision, recall, F_1_-score (the harmonic mean of the precision and recall), and Matthews correlation coefficient (MCC), also revealed the superiority of XGBoost-incremental gene sets compared to the others, as shown in Additional file 1: Figure S1.

Since the gene sets uncovered by the incremental approach did not take into account whether or not the genes were known AMR genes, we also checked the prediction outcomes of the combined gene sets consisting of genes picked by the XGBoost-incremental approach and predicted to be known AMR genes for each of the drug/species combinations. As shown in Fig. 2, the combined gene sets (termed “XGBoost-incremental + Known AMR” in Fig. 2) generally did not outperform genes picked by the incremental approach. The only exception was *E. coli*, in which the combined gene sets achieved an AUROC curve of almost 1.0; however for the three other species, the combined gene sets slightly underperformed the genes selected by the incremental approach.

### Functional analysis of selected gene sets

**Fig. 3:**
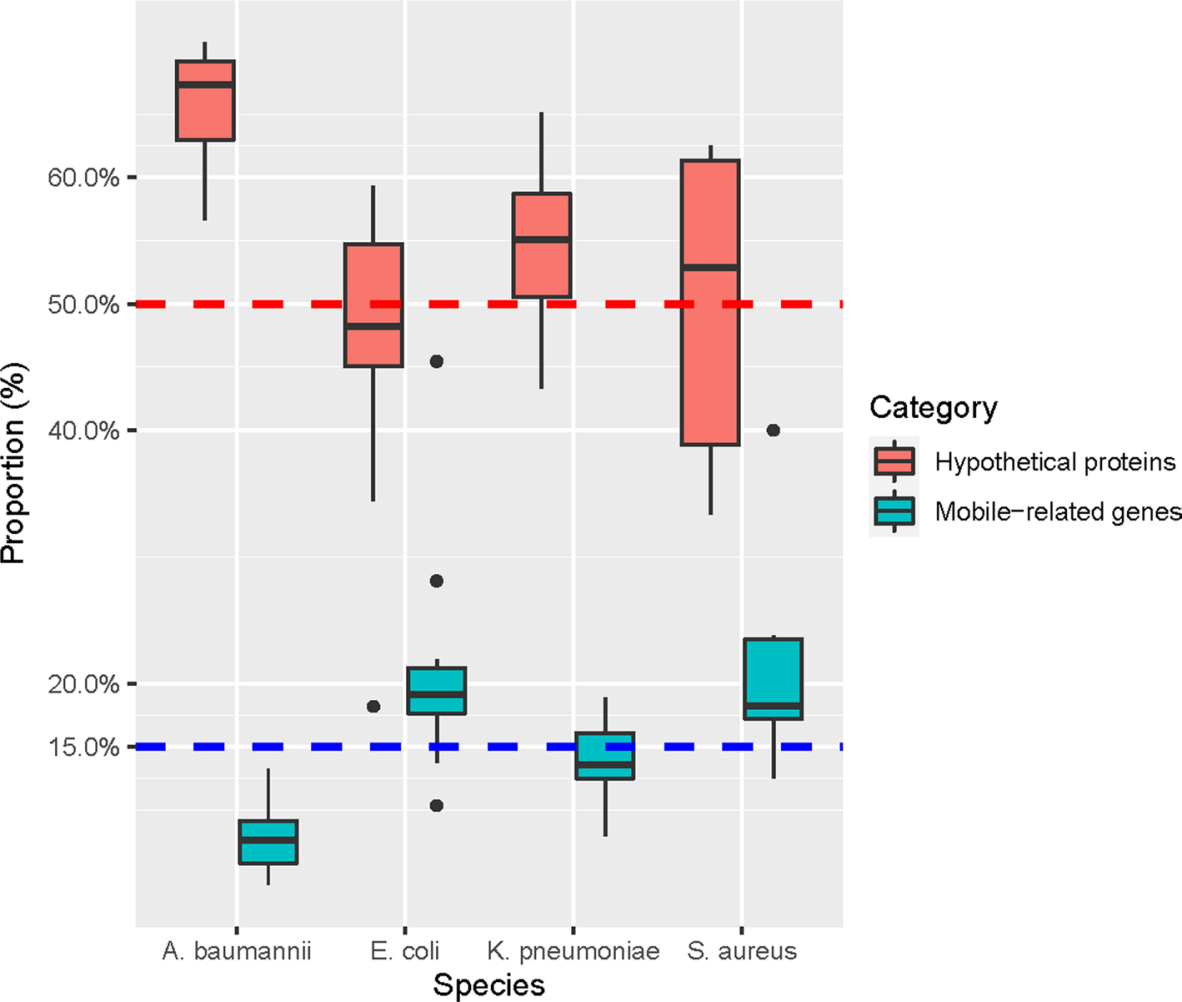
Proportions of hypothetical proteins (i.e., genes with unknown function) and genes related to mobile elements among genes selected using the incremental approach among XGBoost-selected genes. The y-axis is percentages. Reference baselines (50% and 15%) are shown as dashed horizontal lines

We checked the annotations of genes selected by the incremental approach (termed “incremental-genes” or “incremental-gene-set” hereafter) to identify functional roles of those genes. Surprisingly, we found that the majority of incremental-genes were annotated as hypothetical proteins, i.e., genes with unknown functions. As shown in Fig. 3, proportions of hypothetical proteins among the selected genes were generally > 50% for all four species, indicating very high numbers of functionally uncharacterized genes associated with AMR activities. Detailed information can be found in Additional file 1: Table S5. We also checked the proportions of genes related to mobile elements and found that roughly 15% of genes were annotated as having mobile element-related functions, indicating that horizontal gene transfer events or jumping genetic elements may be related to AMR phenotypes.

**Fig. 4:**
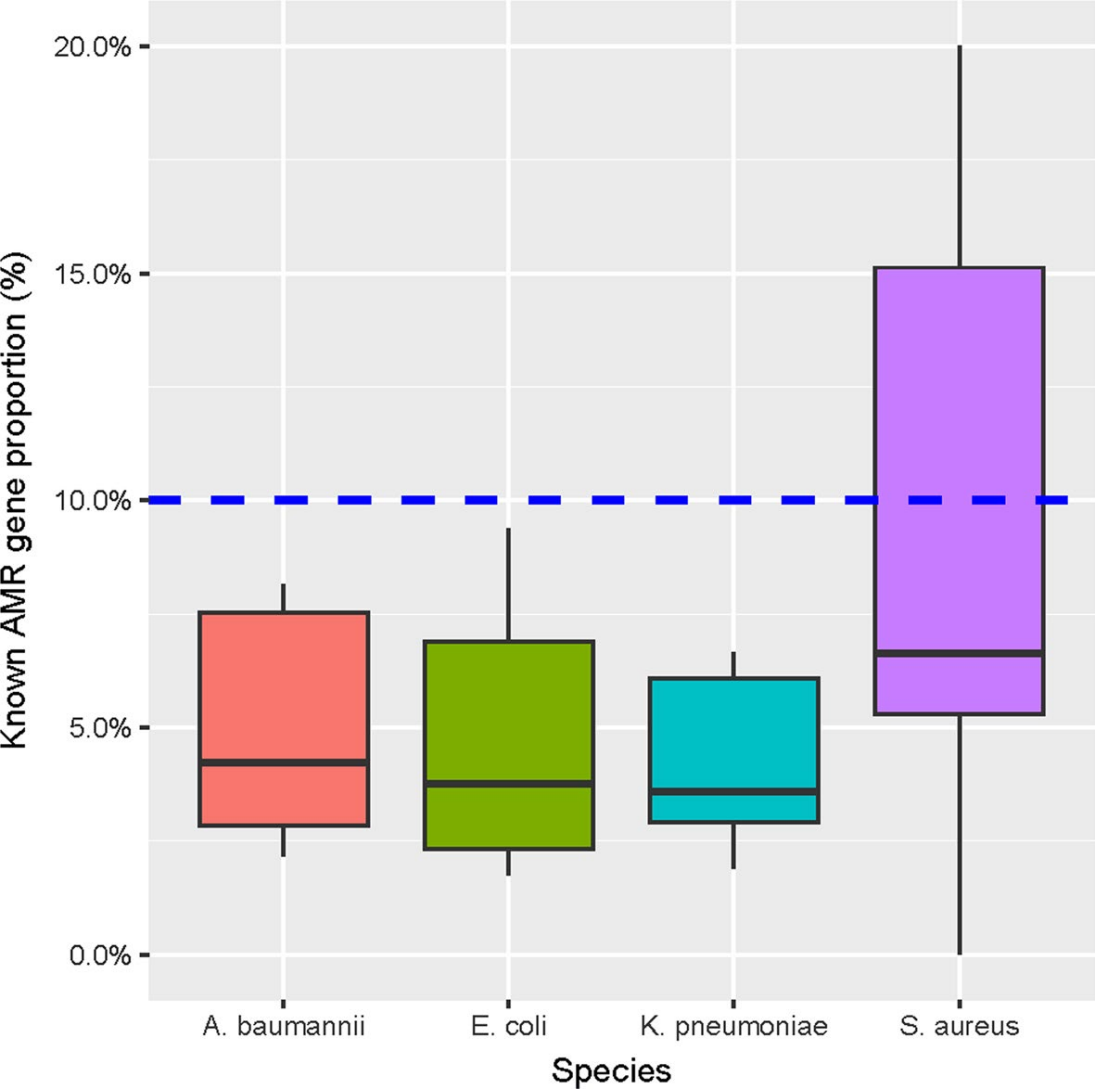
Proportions of known antimicrobial resistance (AMR) genes among genes selected using the incremental approach on the XGBoost-selected genes. Known AMR genes were predicted using CARD/RGI. The y-axis is percentages. A reference baseline (10%) is shown as a dashed horizontal line

By cross-comparing incremental-genes and known AMR genes, we found that the majority of incremental-genes were not known AMR genes. As shown in Fig. 4, proportions of known AMR genes among the incremental-genes were mostly < 10% or even < 5%, indicating that the majority of the genes selected by feature selection approaches were not annotated as known AMR genes. In other words, known AMR genes only accounted for a very small proportion of the genes selected to be highly relevant to AMR phenotypes.

## Discussion

In this study, we attempted to exploit pan-genome gene presence/absence patterns to classify AMR pathogens. By constructing pan-genomes for four bacterial species and building classification models based on those gene presence/absence patterns, we showed that such patterns are indeed capable of classifying antibiotic-resistant pathogens. As shown in Results, simple SVM models built on all gene sets were able to achieve > 80% AUROC levels, indicating that gene presence/absence patterns can be signals representing and predicting whether bacterial pathogens can withstand certain antibiotic drugs.

In the process of building the pan-genomes for the four species, we found that the genetic diversities of *E. coli* and *K. pneumoniae* may be higher than the other two species due to the higher numbers of accessory genes and the elevated Heaps’ law $\gamma$ values of both *E. coli* and *K. pneumoniae*. One may wonder whether the heightened diversities were caused by a representation bias, in which those two species were studied more than the others. We do not think this is the case, as the construction process of pan-genomes was conducted in a de novo manner, in which genes with higher-than-threshold amino acid identities were clustered together. As a result, genetic diversities were only estimated from amino acid sequences without man-made annotations, and whether a species was studied more than the others was not related to the estimated number of accessory genes (i.e., genetic diversity). We also noted that the genome numbers of the four species did not greatly differ (all were between 2000 and 3000; see Table 1 for statistics). Meanwhile, we still did not fully exclude other possibilities that may have contributed to the higher diversities of *E. coli* and *K. pneumoniae*, for example better gene prediction models. Future research may be needed to fully decipher the diversity issue of different bacterial species.

By applying the XGBoost feature selection and an incremental approach to all gene sets, we showed that selecting genes that were more relevant to AMR phenotypes was indeed capable of significantly improving classification outcomes. In addition, genes needed to build classification models were also greatly reduced from tens of thousands to hundreds or even fewer. This result signified the importance of selecting the most relevant genes in probing pathogenic AMR traits.

Comparisons between genes selected by the incremental approach and other gene sets revealed that incremental-genes significantly outperformed both known AMR genes and genes selected by Scoary, a state-of-the-art feature scoring approach. In addition, we also demonstrated that taking into account known AMR genes along with incremental-genes (i.e., the combined gene set of known AMR genes and incremental-genes) did not perform better except in the case of *E. coli*, indicating that incremental-gene-sets are so far the best gene sets that we were able to identify. At the current stage, we do not know the reason why the combined gene sets performed better in *E. coli* but not in others, as neither the AMR database (PATRIC [29]) nor the known AMR gene database (CARD [16]) is tilted toward *E. coli* pathogens. We plan to continue investigating this phenomenon in our ongoing work.

Since the known AMR genes predicted by CARD consisted of drug class information (e.g., penem, cephalosporin, aminoglycoside, etc.), we also checked whether using drug class-specific genes for predicting AMR activities can achieve better performances. By identifying drug classes (e.g., penem, aminoglycoside, tetracycline, etc.) for known AMR genes from the CARD results and specifically picking corresponding genes for each of the drug resistance datasets, we found that class-specific genes generally underperformed compared to all AMR genes (Additional file 1: Figure S2). There may be two reasons for this result. First, drug classes predicted by CARD may be too general in that a CARD annotation might fit into the category of more than one drug. For example, a gene annotated as “beta-lactamase” was annotated as belonging to penem, monobactam, and cephalosporin, as these drugs all belong to the beta-lactam class. Second, machine learning algorithms themselves also have some capabilities in identifying and selecting more-relevant features for model training purposes, thereby avoiding totally unrelated features. However, too many unrelated features may also drag down a model's performance due to noise-handling issues of the classifiers, which is the reason we conducted feature selection on the datasets. We thus reasoned that at the current stage, using all AMR genes instead of drug class-specific genes for prediction purposes could achieve a better performance.

Functionally speaking, genes extracted by the incremental approach consisted of high proportions of unknown functions. Even though proportions of these functionally unknown genes are very high in the bacterial world (can be as high as 98% in the most extreme case [30]), the finding that the majority of potential AMR genes selected by machine learning models belonged to hypothetical proteins was still unexpected. One explanation is that our knowledge of bacterial AMR mechanisms is still far from comprehensive, as also indicated by the low overlapping degree between known AMR genes and incremental genes, and hence this allowed us to unearth many genes with unknown functions using the machine learning feature selection approach. We, however, do not exclude the possibility that some of the incremental-genes selected based on the gene presence/absence patterns are only peripherally related to AMR mechanisms. We noted that this phenomenon was unanimously observed in all four species, indicating that at least some of the hypothetical proteins are worthy of further investigation. We plan to continue investigating these hypothetical proteins in the hope that we can find novel AMR genes and potentially their mechanisms.

On the other hand, one should not be too surprised to see that 10–20% of genes uncovered by the incremental approach were annotated as mobile elements, as previous studies also identified that mobile genetic elements were related to AMR [31–33]. This is because one of the routes for bacterial pathogens to acquire AMR is through horizontal gene transfer, and AMR genes were shown to accumulate on or near mobile elements [34, 35]. Thus, it is not unexpected that mobile elements are related to AMR phenotypes and thus would be selected for predicting AMR pathogens.

By calculating the proportion of hypothetical genes and mobile genetic elements from the bacterial genomes and comparing them against potential AMR genes uncovered by the incremental approach, we found that the distributions were very different between genomic genes and potential AMR genes. As shown in Additional file 1: Tables S6 and S7, the proportions of hypothetical genes and mobile genetic elements identified in potential AMR gene sets were significantly higher than those calculated from all bacterial genomes (*p* values of the Wilcoxon rank sum test were all < 1e−05). This result further indicates that genes with uncharacterized functions may be worth further analyzing in examining their roles in AMR functionalities.

As described in Results, only about 5–10% of the genes selected by the incremental approach were annotated as known AMR genes. We observed that known AMR genes with corresponding resistance mechanisms were very often identified among the AMR genes discovered from the drug resistance datasets. For example, for aminoglycoside resistance [36], genes annotated as “aminoglycoside antibiotic” and “antibiotic inactivation” were found among the extracted AMR genes. Similarly, tetracycline resistance always consisted of genes related to “antibiotic efflux pump,” as the efflux pump is one of the mechanisms bacteria use to withstand tetracycline [37]. Resistance against beta-lactam antibiotics (such as amoxicillin, ampicillin, cefalotin, and ceftazidime, to name just a few) or fluoroquinolones (such as ciprofloxacin) also respectively harbor genes annotated as “beta-lactamase” or “fluoroquinolone resistance”. However, a systematic analysis or comparison of known AMR genes is still prohibitive, since very often the feature selection approach also discovers AMR genes with different resistance mechanisms. This “error” may be due to two reasons: (1) genes with different drug resistance mechanisms are selected since these genes are still weakly related to resistance albeit through different mechanisms, and (2) multiple resistance genes are located nearby and may be carried together by the mobile elements, resulting in statistically significant distinctions. In future work, we will continue seeking approaches to pinpoint known and unknown resistance genes as precisely as we can.

One limitation of this study is that in order to train prediction models, the datasets cannot be too imbalanced, in which we set the criterion as “less than a tenfold difference between the numbers of resistant and susceptible entries”. Even though ensuring more-balanced label proportions is important in building more effective machine learning models, a number of important drugs were also forced to be left out. For example, the ciprofloxacin dataset for *Acinetobacter baumannii* consists of 1035 resistant strains but only 98 susceptible strains, hinting that this drug may very easily encounter drug resistance problems in real life. This dataset however cannot be used to build a highly effective classification model since the model will be highly skewed toward resistant strains and may thus poorly perform compared to more-balanced datasets. In the future we will try using under- or over-sampling approaches to deal with such highly imbalanced datasets and evaluate to what extent can we extract AMR genes from those datasets.

We note that in this study we were looking for gene sets that could be recruited to build highly accurate machine learning models for AMR prediction purposes. Even though the Holy Grail would be the identification of AMR biomarker genes for both prediction and explanatory purposes, the number of genes selected by the XGBoost-based incremental approach is still too many to serve as biomarkers, and many of them are functionally uncharacterized. We however stress that our work not only highlights the importance of gene presence/absence patterns for AMR prediction purposes, but we also unearthed a promising subset of genes for further analyses. Our goal in tackling the AMR prediction problem is to continually shrink gene sets related to AMR mechanisms and devise approaches to identify what the functionally unknown genes are, ultimately achieving the goal of expanding the biomarker repertoire for better elucidation and prediction of AMR pathogens.

## Conclusions

In this study, we showed that the pan-genome-based feature selection approach is able to both select genes most relevant to AMR phenotypes and predict AMR pathogens with very high accuracy. We hope this study can serve as a supplement to conventional known AMR gene-based or SNP-based approaches for better predictions of AMR pathogens.

## Methods

The analytical steps were roughly as follows. Bacterial gene sequences were downloaded and clustered, and pan-genomes were constructed from the clustering results. After extracting presence/absence patterns of gene clusters, machine learning feature selection algorithms were applied to extract gene clusters that were most relevant to the resistance profiles of the antibiotic drugs for these pathogens. The prediction performances of the extracted gene clusters were then evaluated to assess the applicability of these gene clusters for AMR prediction purposes.

### Data collection

Genomic sequences, including genomes (.fna files) and translated proteins (.faa files) for each individual genome of four species (*A. baumannii*, *E. coli*, *K. pneumoniae*, and *S. aureus*) were downloaded from the PATRIC database [29]. Antibiotic resistance profiles of the strains (i.e., whether certain strains were resistant or susceptible to certain antibiotic drugs) were downloaded as well. Genome sequences were searched for their completeness and contamination levels using CheckM v1.1.3 [38] in order to filter out low-quality genomes. 16S ribosomal RNA subunit genes of the NCBI reference genomes of the four species (including *A. baumannii* str. ab736, *E. coli* str. K-12, *K. pneumoniae* subsp. *pneumoniae* HS11286, and *S. aureus* subsp. *aureus* NCTC 8325) were downloaded and searched against the downloaded genomes using NCBI BLASTN [39] (with -max_target_seqs = 1 parameter). Only strains with at least 95% completeness, at most 5% contamination, and at least 99% 16S BLAST identity were retained for further analysis.

### Pan-genome construction

For each species, protein sequences of all strains were collected and clustered using CD-HIT v4.6 [40] at 70% identity. The presence/absence patterns of the yielded gene clusters were constructed for the involved strains in order to build the pan-genome, in which the columns indicate gene clusters while the rows represent different strains. The presence or absence of genes in different strains were checked by looking into the CD-HIT gene clustering results. If, say, genes from strain X were found in gene cluster Y, then we marked the gene cluster as “present” for strain X in the table, and vice versa. In this work we only denoted gene clusters as “present” or “absent” in the tables without considering the number of genes that could be found in each of the clusters.

Core- and accessory-genomes were defined as gene clusters present in all (100%) of the strains or not, respectively. The pan-genome curves were fitted to a Heaps’ law regression growth model ($n = kN^{\gamma }$) according to [41], where *n* is the size of the pan-genome (i.e., the number of gene clusters) and *N* is the number of genomes (strains). Whether the pan-genomes were open- or closed-pan-genomes was based on $\gamma$, in which $\gamma > 0$ indicates an open pan-genome and $\gamma \le 0$ otherwise.

### Selecting antibiotic drugs for prediction

Associations between antibiotic drugs and resistance/susceptible phenotypes were extracted from the file “PATRIC_genomes_AMR.txt” provided by PATRIC [29]. Only strains annotated as “resistant” or “susceptible” were included in our analysis. Information on each drug associated with each species was extracted and merged with the pan-genome tables to form distinct drug tables, in which rows were gene clusters, columns were individual strains, and the table contents consisted of the presence/absence information of gene clusters within the strains along with the drug resistance profiles (i.e., “resistant” or “susceptible”) of the specific drug. In this study separate classifiers were built for different drugs corresponding to different species. For example, different machine learning classifiers were built for predicting “gentamicin resistance of *E. coli* strains,” “gentamicin resistance of *K. pneumoniae* strains,” and “ciprofloxacin resistance of *K. pneumoniae* strains,” to name just a few. Only drugs with (1) at least 100 entries for both resistant or susceptible entries; and (2) less than a tenfold difference between the numbers of resistant and susceptible entries were included in the analysis. A complete list of enrolled drugs and numbers of resistance and susceptible strains for each of the species are provided in Additional file 1: Tables S1–S4. We also note that strains without antibiotic resistance information for specific drugs could not be recruited for prediction purpose and were thus excluded from the corresponding tables.

### Feature selection

Extreme Gradient Boosting (XGBoost) [42] was utilized via the Python XGBoost package (xgboost.XGBClassifier v1.3.0; objective = “binary:logistic”, importantce_type = “gain”, max_depth = 6, and n_estimators = 500) to extract features (gene clusters) relevant to resistant or susceptible phenotypes. All features with > 0 importance values were extracted. Known AMR genes were identified by searching the centroid sequences of gene clusters against the CARD database using its accompanying Resistance Gene Identifier software (RGI v5.0.0) [16]. Scoary was conducted by inputting the gene presence/absence file and phenotype file of each drug into the Scoary GUI interface with default settings [26].

### Machine learning prediction and performance evaluation

An incremental approach was adopted to find a subset of genes among XGBoost-selected genes to achieve even better prediction performances. In general, genes (features) with importance scores evaluated by XGBoost were first sorted into descending order by feature importance values and input, one-by-one cumulatively, into the support vector machine (SVM with a linear kernel) model in an incremental manner. The gene set with the best stratified tenfold cross-validation performance, in which the proportion of labels was preserved in the split sub-datasets, was then selected as the final gene set for the incremental model. The purpose was to find the best gene set (in terms of prediction performance) among all sets of genes. See Additional file 1: Figure S3 for an illustrative example, in which four features with different importance scores were sorted and formed different feature sets in order to find the set with the highest cross-validation prediction performance.

After the incremental gene sets with the best prediction performance were identified, the AMR phenotype prediction performances of the incrementally selected gene sets for each of the drugs were compared against the following feature sets: (1) all gene clusters; (2) known AMR gene clusters predicted by CARD/RGI; (3) gene clusters extracted using Scoary v1.6.16 (with default parameters); and (4) the entire set of XGBoost-selected gene clusters. An additional gene set comprised of the combined set of incrementally selected gene sets and known AMR genes was also added to the comparison for each drug table. The SVM model was constructed and utilized through the Python scikit-learn package [43] with a linear kernel (the regularization parameter C was kept as the default). The predictive performances were also evaluated by stratified tenfold cross-validation and were evaluated by the AUROC curve along with precision, recall, F_1_-score (harmonic mean of precision and recall), and Matthews correlation coefficient (MCC). We noted that the use of the SVM model was selected by comparing the performances against other machine learning algorithms, including a decision tree (with gini impurity for information gain) and random forest (with 100 trees), by applying the algorithms on tenfold stratified cross-validation datasets with the XGBoost-selected feature set. As shown in Additional file 1: Figure S4(A), the SVM and random forest models performed very similarly (i.e., did not significantly differ statistically; Wilcoxon rank sum test *p* values = 0.91, 0.51, 0.54, and 0.28 respectively) while the decision tree model clearly underperformed (*p* ≪ 0.001). In addition, the SVM algorithm was less prone to random effects compared to the random forest, as shown in Additional file 1: Figure S4(B). By running both algorithms repeatedly ten times on the stratified cross-validation dataset and estimating standard deviations of the prediction performances (in terms of AUROC), we identified that results of the SVM algorithm were less variable than those of the random forest model. We therefore selected SVM as the one for incorporation into the incremental model.

### Gene functional annotation

Functional annotations of genes were extracted from the PATRIC database. Genes with “hypothetical protein” annotation were regarded as having unknown functional roles, and genes with the terms including “mobile”, “phage”, “transposase”, “integrase”, or “tail fiber assembly” were classified into mobile-element-related proteins.

## Supplementary Information

**Additional file 1:** Supplementary figures and tables.

**Additional file 2: Table S8**. The PATRIC Genome ID of strains used in building the pan-genome of the four species.

## Data Availability

The analysis was conducted using public data provided by the PATRIC database. The accession numbers (i.e. the PATRIC genome ID) were listed in Additional file 2: Table S8.

## Abbreviations

AMR: Antimicrobial resistance
CARD: Comprehensive Antibiotic Resistance Database
PATRIC: Pathosystems Resource Integration Center
RGI: Resistance Gene Identifier
SVM: Support vector machine
XGBoost: EXtreme Gradient Boosting
