# Supplementary material for: Enhancing predictions of antimicrobial resistance of pathogens by expanding the potential resistance gene repertoire using a pan-genome-based feature selection approach
Source: BMC Bioinformatics. 2022 Apr 15;23(Suppl 4):131. doi: 10.1186/s12859-022-04666-2 (PMC9011928; doi:10.1186/s12859-022-04666-2)
Supplement: Supplementary file 1 — Additional file 1: Supplementary figures and tables. [file 12859_2022_4666_MOESM1_ESM.pdf]

Enhancing predictions of antimicrobial resistance of pathogens by expanding the potential resistance gene repertoire using a pan-genome-based feature selection approach

Ming-Ren Yang and Yu-Wei Wu

Additional Materials

Figures S1-S4

Tables S1-S7

Figure S1

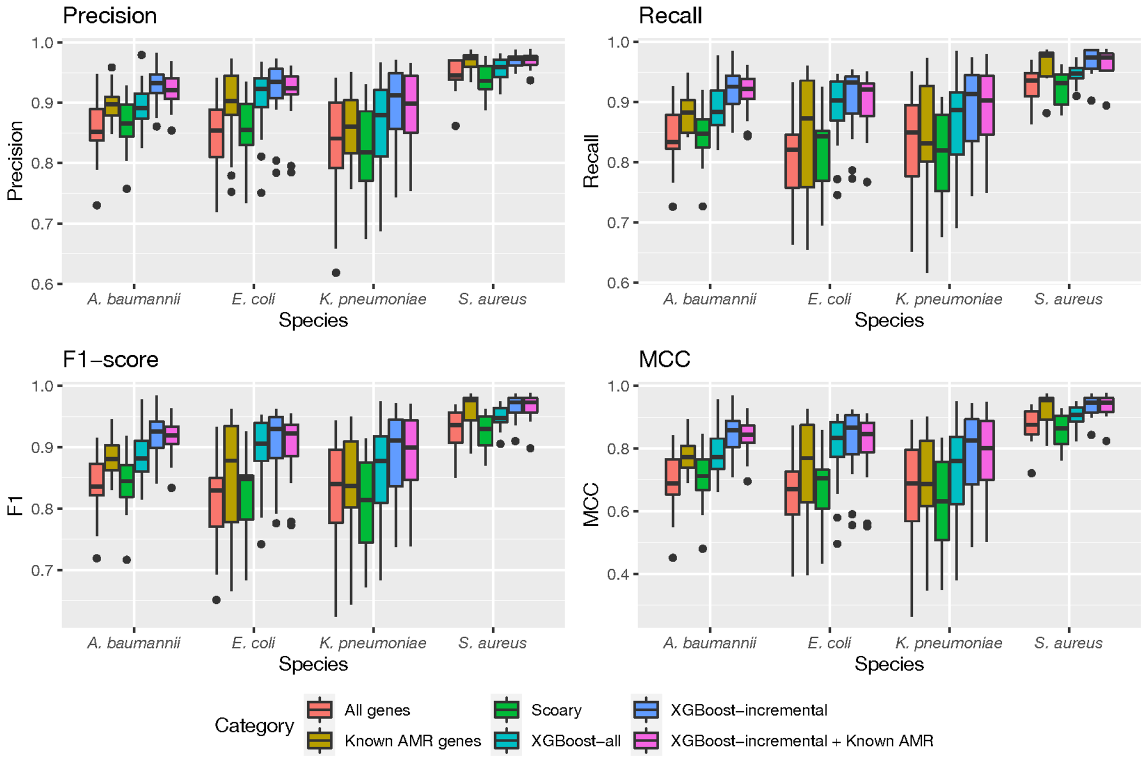

Figure S1. Boxplots indicating different prediction accuracy metrics of different gene sets for antimicrobial resistance (AMR) prediction problems. The metrics include (A) precision, (B) recall, (C) F_1-score, and (D) Matthews correlation coefficient (MCC).

Figure S2

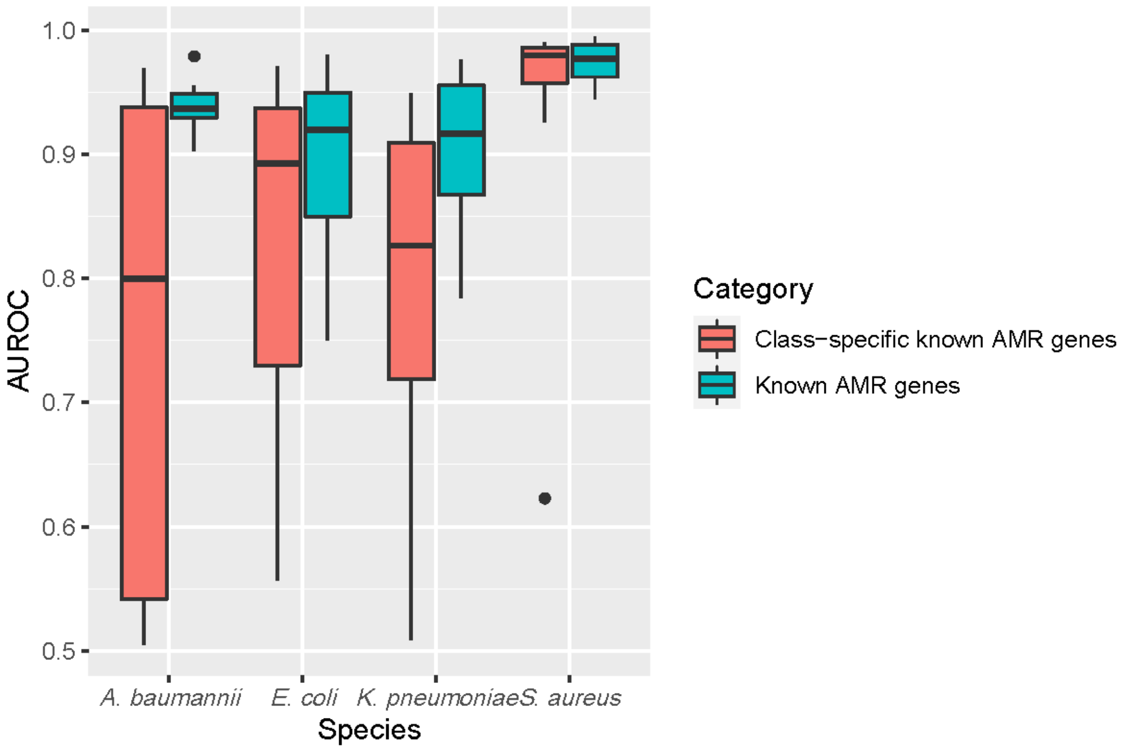

Figure S2. The prediction performance comparison between using all AMR genes and antibiotic-class-specific AMR genes. Y-axis indicates the prediction performance in terms of Area Under Receiver Operating Characteristics (AUROC) curve.

Figure S3

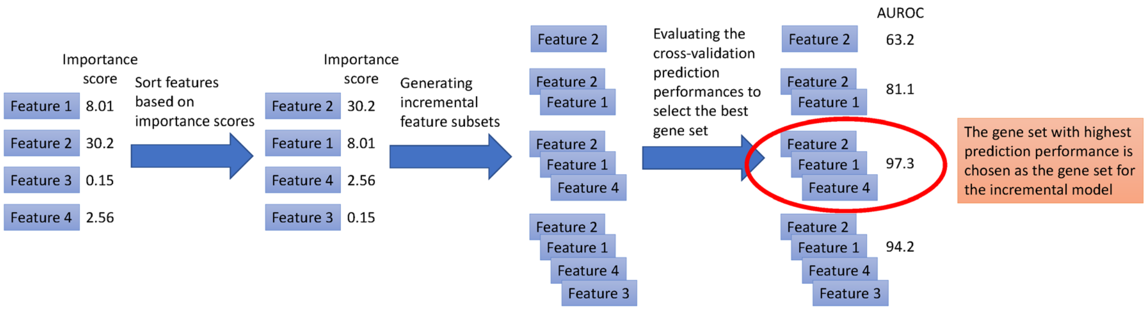

Figure S3. An illustrating example showing how the incremental approach works. Given four features with different importance scores (evaluated by XGBoost), the features are first sorted into descending order, and the incremental approach is going to start from an empty set and gradually add features, one by one, into the empty set and evaluate the prediction performances the current feature set using cross validation. The feature set with the highest prediction performance is then extracted and output as the feature set for the incremental model.

Figure S4

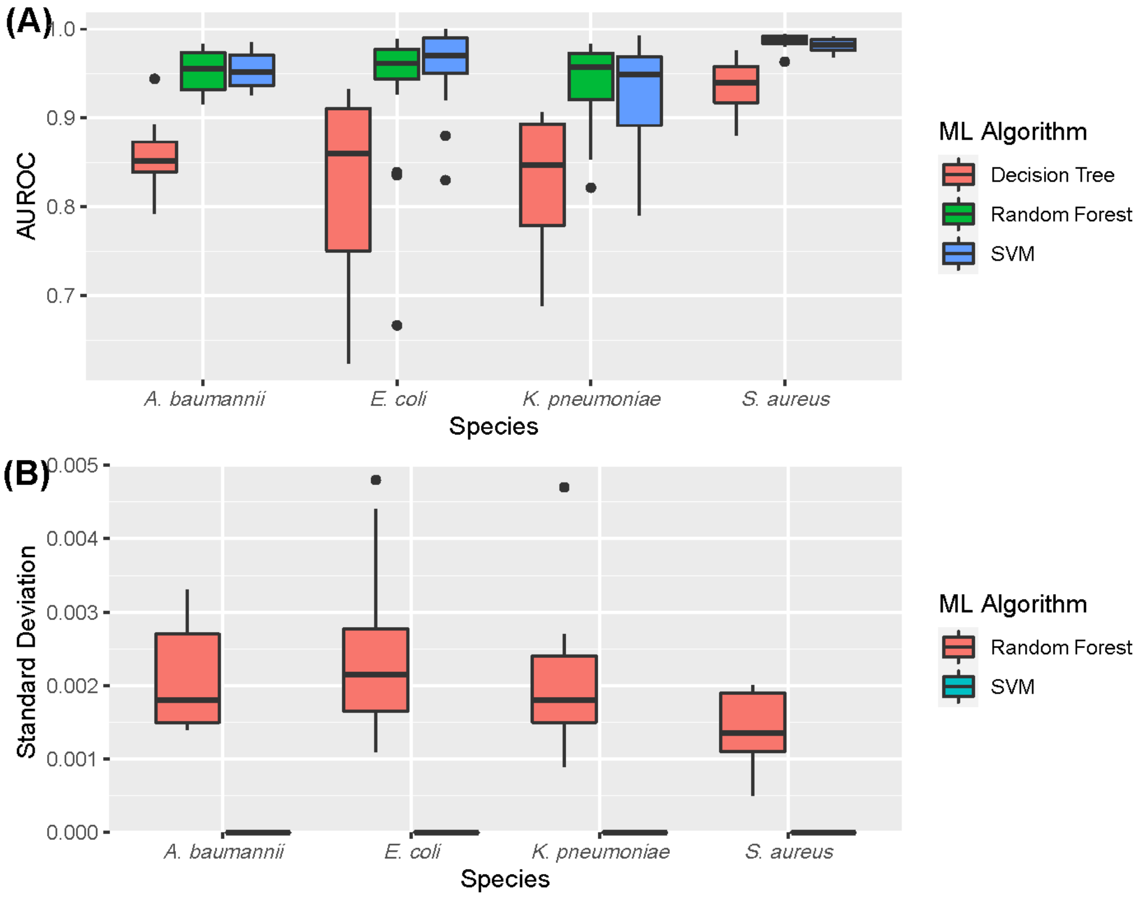

Figure S4. Comparison of different machine learning algorithm performances for the incremental model. (A) Boxplot indicates the prediction performances of different algorithms, including SVM, decision tree and random forest; (B) standard deviation distribution of ten repeated model runs for SVM and random forest.

Table S1. Numbers of resistant and susceptible strains for the selected drugs of *Acinetobacter baumannii*. Yellow entries indicate the drug datasets analyzed in this study.

Antibiotic drugs	Resistant	Susceptible
Imipenem	595	635
ciprofloxacin	1035	98
gentamicin	996	100
amikacin	580	461
ceftazidime	858	144
trimethoprim/sulfamethoxazole	835	121
tobramycin	658	296
tetracycline	710	198
ampicillin/sulbactam	464	421
ceftriaxone	777	57
levofloxacin	671	153
ampicillin	799	1
cefotaxime	755	29
aztreonam	768	2
cefazolin	717	0
nitrofurantoin	696	0
meropenem	366	204
cefoxitin	503	0
cefepime	316	18
tigecycline	6	212
doripenem	61	152
piperacillin/tazobactam	189	0
carbapenem	105	60
ticarcillin/clavulanic acid	134	3
colistin	13	117
cefotetan	112	0
moxifloxacin	60	31
polymyxin B	8	78
minocycline	17	56
ertapenem	71	0
azidothymidine	49	0
piperacillin	48	0
ticarcillin	46	0
timentin	34	4
amoxicillin/clavulanic acid	27	0
kanamycin	24	0
sulphonamides	24	0
streptomycin	23	0
spectinomycin	22	0
nalidixic acid	21	0

neomycin	21	0
trimethoprim	20	0
cefuroxime	6	0
chloramphenicol	3	0
netilmicin	3	0
azithromycin	1	0
benzylpenicillin	1	0
clarithromycin	1	0
daptomycin	1	0
erythromycin	1	0
fosfomycin	1	0
linezolid	1	0
rifampin	1	0
teicoplanin	1	0
vancomycin	1	0

Table S2. Numbers of resistant and susceptible strains for the selected drugs of *Escherichia coli*. Yellow entries indicate the drug datasets analyzed in this study.

Antibiotic drugs	Resistant	Susceptible
ciprofloxacin	530	1460
gentamicin	222	1735
ceftazidime	256	1632
amoxicillin/clavulanic acid	570	1148
piperacillin/tazobactam	154	1535
tigecycline	2	1537
cefotaxime	205	1326
cefuroxime	245	1266
imipenem	42	1214
amoxicillin	663	434
ampicillin	707	238
meropenem	57	820
amikacin	27	842
cefepime	135	700
tobramycin	124	654
ertapenem	63	634
cefoxitin	112	477
trimethoprim	259	320
aztreonam	159	409
ceftriaxone	292	201
tetracycline	276	179
trimethoprim/sulfamethoxazole	192	103
levofloxacin	217	66
nitrofurantoin	13	268
cefalotin	60	191
chloramphenicol	50	194
cefazolin	208	13
streptomycin	93	98
azithromycin	16	156
sulfamethoxazole	112	58
norfloxacin	28	136
ampicillin/sulbactam	83	9
doripenem	24	60
nalidixic acid	28	22
cephalothin	43	2
ceftiofur	41	1
sulfisoxazole	35	1
cefazoline	15	11
gentamycin	7	19
sulfamethoxazole/trimethoprim	7	19
colistin	3	14

ceftazidime/avibactam	4	11
Fosfomycin	2	10
ceftolozane/tazobactam	3	4
kanamycin	1	6
moxifloxacin	5	2
doxycycline	2	4
minocycline	2	4
piperacillin	4	2
augmentin	4	0
chloram	0	4
nalidixate	4	0
sulfixazole	4	0
tms	4	0
cefotetan	0	3
ceftaroline	3	0
ticarcillin/clavulanic acid	2	1
cefotaxime/clavulanic acid	2	0
ticarcillin	1	1
benzylpenicillin	1	0
cefalexin	1	0
cephalexin	1	0
clarithromycin	1	0
clindamycin	1	0
daptomycin	1	0
erythromycin	1	0
linezolid	1	0
netilmicin	1	0
rifampin	1	0
sulbactam	1	0
teicoplanin	1	0
temocillin	1	0
vancomycin	1	0

Table S3. Numbers of resistant and susceptible strains for the selected drugs of *Klebsiella pneumoniae*. Yellow entries indicate the drug datasets analyzed in this study.

Antibiotic drugs	Resistant	Susceptible
ciprofloxacin	1993	351
trimethoprim/sulfamethoxazole	1764	579
gentamicin	990	1293
meropenem	855	1385
ceftazidime	1987	166
ampicillin	2082	4
cefazolin	1892	187
ceftriaxone	1896	175
imipenem	818	1240
amikacin	222	1810
levofloxacin	1636	389
aztreonam	1718	226
cefoxitin	1108	824
piperacillin/tazobactam	1354	549
tobramycin	1111	750
cefepime	1237	581
tetracycline	898	837
ampicillin/sulbactam	1594	91
cefuroxime/sodium	1449	91
nitrofurantoin	811	92
ertapenem	497	94
cefotaxime	421	6
amoxicillin/clavulanic acid	291	119
tigecycline	19	320
doripenem	218	27
ticarcillin/clavulanic acid	75	92
trimethoprim	60	96
ceftazidine	20	92
norfloxacin	13	96
colistin	31	74
cetotaxime	92	4
cephalothin	73	5
ofloxacin	44	27
chloramphenicol	40	20
polymyxin B	8	48
cefuroxime	50	4
cefalotin	44	1
doxycycline	6	35
minocycline	4	31
cefotaxime/clavulanic acid	24	0
fosfomycin	13	1

amoxicillin/clavulanate	12	0
florfenicol	10	2
ceftazidime-avibactam	2	7
moxifloxacin	7	0
piperacillin	6	1
cefotetan	2	3
ceftolozane-tazobactam	4	0
cefalexin	3	0
sulbactam	3	0
amoxicillin	2	0
azithromycin	2	0
benzylpenicillin	2	0
ceftaroline	2	0
clarithromycin	2	0
daptomycin	2	0
erythromycin	2	0
linezolid	2	0
rifampin	2	0
sulfamethoxazole	2	0
teicoplanin	2	0
vancomycin	2	0
clindamycin	1	0

Table S4. Numbers of resistant and susceptible strains for the selected drugs of *Staphylococcus aureus*. Yellow entries indicate the drug datasets analyzed in this study.

Antibiotic drugs	R	S
methicillin	735	879
gentamicin	172	1410
erythromycin	519	1060
penicillin	1131	178
tetracycline	206	1058
ciprofloxacin	467	765
fusidic acid	83	1144
rifampin	23	1134
vancomycin	0	1150
clindamycin	384	522
trimethoprim/sulfamethoxazole	169	434
mupirocin	7	537
trimethoprim	15	499
oxacillin	63	305
levofloxacin	31	279
cefoxitin	34	246
fosfomycin	1	249
linezolid	0	91
doxycycline	1	49
daptomycin	1	36
ceftaroline	1	29
amoxicillin/clavulanic acid	10	9
chloramphenicol	1	13
ampicillin	9	0
ampicillin/sulbactam	2	7
cefazolin	2	7
ceftriaxone	2	7
moxifloxacin	0	9
cephalothin	7	0
imipenem	0	7
meropenem	0	7
ofloxacin	3	4
tigecycline	0	5
minocycline	0	4
teicoplanin	0	3
nitrofurantoin	0	2
dicloxacillin	1	0
quinupristin/dalfopristin	0	1
tobramycin	1	0

Table S5. Proportion of hypothetical proteins and mobile element-related genes of those selected using the incremental gene selection method on top of XGBoost feature selection results.

Species	Drug	Hypothetical proteins	Mobile-related genes
Escherichia coli	amoxicillin	47.5%	20.5%
	amoxicillin/clavulanic acid	48.0%	19.1%
	ampicillin	45.1%	18.6%
	aztreonam	18.2%	45.5%
	cefalotin	58.6%	10.3%
	cefepime	52.9%	17.6%
	cefotaxime	51.9%	13.8%
	cefoxitin	48.2%	18.0%
	ceftazidime	50.9%	19.1%
	cefuroxime	54.7%	21.8%
	ciprofloxacin	45.1%	21.2%
	gentamicin	34.4%	28.1%
	levofloxacin	39.9%	21.9%
	tetracycline	59.3%	16.9%
	tobramycin	56.4%	14.9%
	trimethoprim	54.9%	19.7%
	trimethoprim/sulfamethoxazole	44.7%	20.1%
Acinetobacter baumannii	amikacin	62.5%	8.8%
	ampicillin/sulbactam	67.3%	6.3%
	ceftazidime	70.7%	9.2%
	gentamicin	70.6%	5.6%
	imipenem	67.3%	4.1%
	levofloxacin	68.5%	7.7%
	meropenem	69.4%	5.6%
	tetracycline	64.3%	13.2%
	tobramycin	59.0%	7.6%
	trimethoprim/sulfamethoxazole	56.6%	13.2%
Klebsiella pneumoniae	amikacin	65.1%	10.4%
	amoxicillin/clavulanic acid	43.3%	17.5%
	aztreonam	58.6%	15.7%
	cefepime	61.9%	12.5%
	cefoxitin	50.7%	13.2%
	ciprofloxacin	57.5%	13.2%
	gentamicin	58.7%	7.9%
	imipenem	48.5%	17.6%
	levofloxacin	48.1%	18.9%
	meropenem	60.4%	12.5%
	piperacillin/tazobactam	54.7%	14.7%

	tetracycline	55.4%	13.9%
	tobramycin	50.5%	16.2%
	trimethoprim/sulfamethoxazole	54.2%	10.6%
Staphylococcus aureus	ciprofloxacin	57.4%	23.4%
	clindamycin	33.3%	23.8%
	erythromycin	35.3%	17.6%
	gentamicin	40.0%	40.0%
	methicillin	62.5%	12.5%
	penicillin	48.3%	17.2%
	tetracycline	62.5%	18.8%
	trimethoprim/sulfamethoxazole	61.0%	17.1%

Table S6. Distribution of hypothetical gene proportion for bacterial genomes and potential AMR resistance genes identified by the incremental approach.

Species	Mean (std) of bacterial genomes	Mean (std) of potential AMR genes	Wilcoxon rank sum test p-value
Acinetobacter baumannii	0.26 (0.02)	0.64 (0.04)	4.844e-10
Escherichia coli	0.13 (0.02)	0.48 (0.10)	1.199e-12
Klebsiella pneumoniae	0.16 (0.02)	0.56 (0.06)	1.183e-14
Staphylococcus aureus	0.21 (0.01)	0.50 (0.12)	2.164e-07

Table S7. Distribution of mobile-related gene proportion for bacterial genomes and potential AMR resistance genes identified by the incremental approach.

Species	Mean (std) of bacterial genomes	Mean (std) of potential AMR genes	Wilcoxon rank sum test p-value
Acinetobacter baumannii	0.02 (0.01)	0.08 (0.03)	6.956e-10
Escherichia coli	0.05 (0.02)	0.20 (0.08)	1.157e-13
Klebsiella pneumoniae	0.03 (0.01)	0.13 (0.03)	1.183e-14
Staphylococcus aureus	0.05 (0.02)	0.22 (0.08)	2.164e-07
